# Timing of malaria messages for target audience on radio airwaves

**DOI:** 10.1186/1475-2875-11-283

**Published:** 2012-08-20

**Authors:** Vincent Batwala, Pascal Magnussen, Justine Mirembe, Edgar Mulogo, Fred Nuwaha

**Affiliations:** 1 Department of Community Health, Mbarara University of Science & Technology, PO Box 1410, Mbarara, Uganda; 2 Centre for Health Research and Development, Faculty of Life Sciences, Copenhagen University, Thorvaldsensvej 57, DK1871, Frederiksberg C, Denmark; 3 The AIDS Support Organisation (TASO), Mbale Centre, PO Box 2250, Mbale, Uganda; 4 Disease Control and Environmental Health, Makerere University School of Public Health, PO Box 7072, Kampala, Uganda

## Abstract

**Background:**

Due to the limitations of face-to-face communication to teach families how to manage, control and prevent malaria, national and local malaria programmes try to reach people through the radio. However, information regarding the timing of radio messages for the target audiences is lacking.

**Methods:**

Within a large-scale trial (Clinicaltrials.gov: NCT00565071), data regarding the time at which people listen to the radio was collected from 1,628 consenting outpatients (and caregivers for minors) attending six rural government primary level health care centres in Bushenyi and Iganga districts of Uganda from February to July 2011.

**Results:**

The majority of households, 1,099 (67.5%) owned a radio. The majority, 1,221 (86.3%), participants had heard about malaria from the radio. Some participants started listening to the radio at about 06.00 East African local time (EAT). The peak hours at which people listen to the radio are 12.00-14.00 and 18.00-23.00 local time. The median time of listening to the radio by men is 20.00 (inter-quartile range (IQR): 18.30-21.00) and women 19.30 (IQR: 13.00-20.30).

**Conclusion:**

Planners of malaria radio interventions need to broadcast their messages within the two peak EAT of 12.00-14.00 and 18.00-23.00.

## Background

Communication is an important tool to help communities learn ways to prevent and treat malaria [1], a disease which caused an estimated 655,000 deaths in 2010 [2]. In sub-Saharan Africa, malaria mostly kills children under five years of age and pregnant women, while men are often the key to deciding whether, when and where to seek care.

Face-to-face communication is still the single most effective method of teaching families how to manage, control and prevent malaria. In this regard, communication materials have been developed on how health workers should advise patients and caretakers of young children. This communication methodology is now incorporated into clinical training programmes. However, face-to-face communication has limitations: the advice given by health workers reaches only those families who already come to health facilities, outreach posts and during home visiting by health workers. For various reasons (distance, time, money for transport, preference for other types of treatment), some people do not come to health facilities and thus miss out on important health messages. Even those who visit health units spend less than the recommended consultation time with health care providers. Communicating effectively under this circumstance presents an enormous challenge and explains the poor provider-client interaction [3]. National malaria programmes try to reach these people through local radio programmes (radio talk-shows, radio spots, drama, etc.) as part of an overall campaign to help improve the effectiveness of malaria interventions [4]. Therefore, the radio is an influential communication channel that reaches more people in their homes than any other mass medium. It is a channel for giving new information, and for reinforcing what families may have learned from health workers. It helps motivate people to act through entertainment and compelling presentations [1,4].

With 112 districts, Uganda has 275 Frequency Modulation (FM) radio stations [5]. Thus the radio is the communication tool most available and the rural population rely heavily on it to stay informed. Much of the radio industry is regionally based, with broadcasts in local languages and programming that caters to local tastes [6]. People access radios at home, in cars, with commercial motorcyclists (locally known as *Boda boda*), on mobile telephones, in restaurants and bars. Radios are even carried in order to listen while tilling gardens. Radio programmes are normally of long duration and listeners can call-in with their mobile phones for enrichment. The radio though is not a direct, interactive dialogue, such as when talking to others face-to-face. But well developed, targeted radio programmes do facilitate the transfer and uptake of messages. Therefore, harnessing the power of the radio to promote behaviour change can be easy when you know how. The steps for developing effective radio messages (radio spots, for example) have been described previously [1,4]. However, information regarding the timing of messages for the target audiences is lacking. This paper assessed the appropriate timing for malaria messages through the radio airwaves.

## Methods

### Study design and setting

The study design was cross sectional. Data was collected as part of a large-scale trial (Clinicaltrials.gov: NCT00565071) from February to July 2011 in Bushenyi and Iganga districts of Uganda. The two districts were selected through a stratified random selection technique. Stratification was based on the level of transmission intensity. Bushenyi has low malaria transmission while Iganga has high malaria transmission intensity. The annual entomological inoculation rates are not known, but in the nearby Kanungu and Tororo districts are reported to be <10 and >500 infective bites per person per year respectively [7]. The study was carried out before the two districts were partitioned. The detailed description of the study setting is given elsewhere [8], but briefly as shown below.

### Bushenyi district

Bushenyi has a population of 731392. The district is divided into seven health service zones (health sub-districts or HSDs) with four hospitals, six health centres (HC) at county level, 20 government HCs at sub-county level and 56 HCs at parish level. The district experiences low and unstable malaria transmission, with people of all ages being at risk. It is epidemic-prone, with occasional malaria outbreaks occurring shortly after the rains. With a total land area of 3,949 sq km, the district is endowed with diverse natural resources that include arable land, forests, large lake water bodies (Edward, George and Kazinga Channel), wetlands, rivers (Kyambura, Nchwera, Kaizi and Rwempunu), Queen Elizabeth National Park and minerals. The main economic activities are semi-intensive agriculture (growing crops and rearing animals), fishing and trade. The district is multi-ethnic with varying customs and norms. The main inhabitants are *Banyankore* and *Bakiga*[9].

### Iganga district

Iganga has a total population of 540,939 with an average growth rate of 3.4% and population density of 322 persons per sq km. The district is divided into four HSDs. It has one public hospital, three county level HCs, 15 sub-county level government HCs and 57 HCs at parish level. Malaria is the leading cause of morbidity, and outpatient attendance for all age groups. The population is ethnically heterogeneous but the indigenous inhabitants are *Basoga*. Subsistence farming is the main economic activity followed by small-scale trade (shop and market vendors) [10].

### Study population and sample size estimation

Information was collected from 1,628 outpatients (and caregivers of minors) attending six rural sub-county government HCs (three HCs in each district). This sample size was estimated using a standard formula [11], assuming 50% of the rural population listen to the radio, alpha error of 5% and adjustment for stratification and non-response.

### Enrolment

Outpatients presenting with fever (by statement or measured axillary temperature ≥37.5 °C) and suspected by clinicians to be suffering from malaria were informed about the study and requested to sign an informed consent form. The study participants were consecutively enrolled at each of the HCs. Data was collected using a pre-tested, interviewer-administered, semi-structured questionnaire. To enhance privacy and confidentiality, one-to-one interviews were conducted in a separate room. The specific questions that were the focus for this paper were: 1) Does your household have a radio? 2) Do you listen to the radio? 3) At what time(s) do you listen to the radio? 4) Have you ever heard about malaria? 5) From where did you hear about malaria (mention all sources of malaria information)? Questions 3 and 5 were open-ended.

### Data management

The filled questionnaires were checked manually for completeness and un-coded data were coded before entry in EpiData version 3.1 (EpiData Ass. Odense, Denmark). Analysis was carried out in STATA version 11 and Microsoft Excel.

### Ethical considerations

The study was approved by Makerere University School of Public Health Institutional Review Board; and the Uganda National Council for Science and Technology (Ref: HS 209). Written informed consent was sought from participants at the time of interview. The main trial was registered with the Clinicaltrials.gov (NCT00565071).

## Results

### Sociodemographic and economic profile of participants

A total of 1,628 participants were enrolled. Each district had equal contribution to the sample. Some 207 (12.7%) came from female-headed households. The median number of people in the household was six (inter-quartile range (IQR): 4–7). The median monthly household income was US$8 (IQR: 4.0-19.9). Less than half (733 or 45.0%) slept under a mosquito bed net the night before data collection for this study. The median duration of sleeping under the mosquito bed net was five months (IQR: 3–12). The median distance in kilometres from home to the HCs was 3.2 (IQR: 1.6-4.8). About 58 (3.6%) participants paid for transport to come to HCs. The mean transport fee (one-way) was US$0.46 (95%CI: 0.36-0.56). Table 1 shows additional sociodemographic and economic characteristics of study participants.

**Table 1:** Selected sociodemographic and economic characteristics of participants

**Variable**	**n (%)**
Staying in personal house	1,596 (98.0)
Type of house	
Temporary	420 (25.8)
Semi-permanent	842 (51.7)
Permanent	366 (22.5)
Household assets	
Radio	1,099 (67.5)
Television	28 (1.7)
Bicycle	754 (46.3)
Motorcycle	169 (10.4)
Car	93 (5.7)
Electricity in house	39 (2.4)
Source of water for use*	
Harvest rain water	1,193 (73.3)
Protected well	1,099 (67.5)
Borehole	730 (44.8)
Tap (running) water	80 (4.9)
Main transport to health centre	
Walked	1,249 (76.7)
Bicycle	335 (20.6)
Motorcycle (*Boda boda*)	24 (1.5)
Taxi	20 (1.2)
Ever heard about malaria*	1,406 (86.4)
From radio	1,221 (86.8)
From neighbour/friend	1,154 (81.1)
From health worker	1,088 (77.4)
From print media	309 (22.0)

*participant mentioned more than one option.

### Time of listening to radio

**Figure 1:**
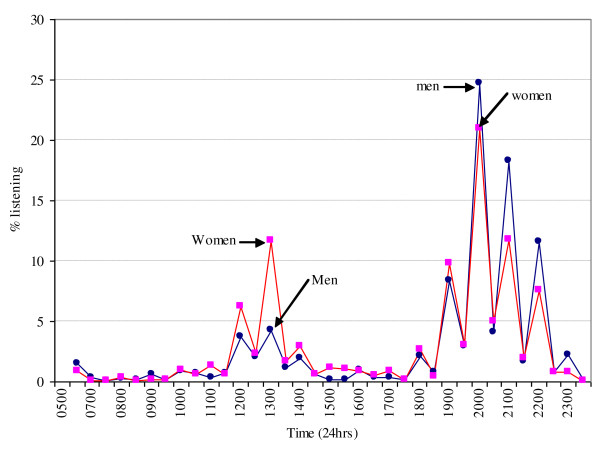
Time of listening to radio.

The majority of households, 1,099 (67.5%), owned a radio (Table 1). This included 505 (62.0%) in Bushenyi and 594 (73.4%) in Iganga districts. Overall, of 529 participants from households that did not own radios, 61 (11.5%) listened to the radio. Generally, 1,221 (86.3%) had heard about malaria from the radio. Some participants start listening early at about 06.00 East African local time. However, listening to the radio peaks between 12.00-14.00 and a second peak between 18.00-23.00 local time (Figure 1). The median time of listening to the radio by men is 20.00 (IQR: 18.30-21.00) and women 19.30 (IQR: 13.00-20.30) (Table 2). The tendency to listen to the radio did not differ across age groups (14–17, 18–29 and 30+ years), and mirrored the aforementioned peak hours.

**Table 2:** East African local time of listening to radio

**Target audience**	**Median (IQR)**
Men	20.00 (18.30-21.00)
Women	19.30 (13.00-20.30)

Time (in 24-hour clock), IQR = inter-quartile range.

## Discussion

Broadcasting important health messages on the radio to target specific audiences has been challenging. The practice is to broadcast messages at any time during the day and sometimes up to 24.00 at night. For example in eastern Uganda, a radio spot advocating the treatment of malaria using artemisinin-based combination therapy (ACT) and to enhance adherence to the drug is usually broadcast between 09.00-10.00 and 16.00-18.00. Further, a non-governmental organization caring for people living with the human immunodeficiency virus (HIV) in the same region of Uganda runs a radio talk-show intervention programme every Saturday from 08.00-10.00 at a cost of US$28 per hour. However, results from this study suggest that planners of health interventions intending to integrate the radio as a medium of communication to increase their effectiveness should critically focus on the appropriate time at which an adequate proportion of the target audience is likely to be listening.

Although the proportion of people listening may vary depending on their favourite programmes, results from the current study show that there are generally two peaks at which people listen to the radio. More rural women listen to the radio than men between 12.00-14.00 although the proportion of those listening is below 15%. The highest peaks are between 18.00-23.00 where up to 21% and 25% of women and men respectively listen to the radio. Further to this, more men than women listen during later hours tapering off at 23.00 before retiring to bed. These peak hours should be the focus for broadcasting malaria messages. However, there are budgetary implications here as companies intending to advertise products are likely to compete for the same peak hours. Therefore, planners of malaria and other health-related radio interventions need to be generous in budgeting for the airtime at these peak hours.

It is a waste of the scarce resources to buy radio airtime and broadcast messages between midnight and 05.00 because people are sleeping. Further, the proportion of people listening between 06.00-11.00, and just after 14.00 to just before 18.00 is less than four percent and thus not attractive for investment.

Neighbours and/or friends are useful resources for malaria information as reported here (81.6% obtained malaria information from neighbours/friends). This implies that even those who do not have radios or have missed malaria intervention radio programmes are likely to benefit through sharing such information by way of family and social networks. The information from health workers also trickles down to those who do not attend health facilities through sharing across social networks. Sharing information helps to hone key points heard from the radio or from health workers.

One constraint of this paper was that the sample consisted of patients already attending health facilities. This sample may exhibit different healthcare-seeking behaviour compared to those people remaining in the community. However, although this is a likely scenario, the tendency to listen to the radio may not significantly differ as messages find them all at home or places where they normally listen to the radio. In addition, it might just be that those were the patients at that time, but it may be the majority of those who remained in the community were also patients attending health centres at other points in time. This study was carried out among the rural populations. In urban areas there is currently a movement towards “lunch-hour” prayers that at times extend from 11.00-14.00 local time. This is likely to impact on the 12.00-14.00 peak listening hours reported here, and thus is an area for further inquiry.

This paper carries a simple straight forward message based on data, which identify the peak times of listening to the radio and argues that these are the optimal times that malaria messages should be broadcast. The paper also identifies examples of actual broadcast time when few people are listening. This inefficiency of reaching the broader audience is not particular to malaria only, but also to other health problems. Both the lack of adaptation to the optimal broadcast times as well as the possible competition with other commercial programs thus leading to higher cost of air time are mentioned. The radio knowledge reported here has not been available. However, further inquiry is needed to clarify if higher radio costs may be acceptable relative to other interventions towards malaria and other health problems.

## Conclusion

Planners of malaria radio interventions need to broadcast their messages within the two peak times of 12.00-14.00 and 18.00-23.00.

## Competing interests

The authors declare that they have no competing interests.

## Authors’ contributions

All authors conceived and designed the study; VB and FN collected, analysed, interpreted the data and drafted the manuscript; PM, JM and EM critically revised the manuscript. All authors read and approved the final manuscript.
